# Alleviating mitochondrial dysfunction in diabetic cardiomyopathy through the Adipsin and Irak2 pathways

**DOI:** 10.1186/s40779-024-00513-y

**Published:** 2024-02-01

**Authors:** Mabel L. Cummins, Grace Delmonte, Skylar Wechsler, Joseph J. Schlesinger

**Affiliations:** 1 Department of Anesthesiology, Vanderbilt University Medical Center, Nashville, TN 37212 USA; 2 Division of Critical Care Medicine, Department of Anesthesiology, Vanderbilt University Medical Center, Nashville, TN 37212 USA

**Keywords:** Diabetic cardiomyopathy, Mitochondrial function, Lipotoxicity, Cardiomyocytes, Interleukin-1 receptor-associated kinase-like 2 (Irak2), Interleukin-1, Protein kinase R

Diabetic cardiomyopathy (DCM) is a major cause of heart failure in diabetic patients. It progresses asymptomatically prior to the onset of severe cardiac symptoms [1]; therefore, elucidating the underlying mechanisms of DCM is critical to providing early treatment options. This commentary elaborates on the findings of Jiang et al. [2], who investigated the role of adipokine hormone, Adipsin, as a cardioprotective factor in DCM. We provide an exposition and alternative treatment considerations, like Fisetin, and discuss the potential of investigating other cellular targets implicated in cardiac dysfunction, like the interleukin-1 receptor-associated kinase-like 2 (Irak2) protein [3] and protein kinase R [4].

Elevated circulation of fatty acids (FAs) in diabetes leads to their ectopic accumulation in other organs, like the heart. This accumulation causes lipotoxicity, exacerbates oxidative stress and leads to cell and organ dysfunction [1]. When the excess utilization and uptake of lipids exceeds the adaptation of the heart, myocardial contractile function decreases, leading to heart failure. A treatment to reverse myocardial lipotoxicity and treat damaged mitochondrial tissue is currently unknown [5]. However, Adipsin (an adipokine implicated in poor cardiovascular function resulting from diabetes mellitus) may regulate myocardial metabolism and function.

Jiang et al. [2] measured cardiac function, lipid accumulation, and FAs oxidation in myocardial cells treated with Adipsin overexpression or *Irak2* knockdown (a protein downstream of Adipsin). Adipsin is a metabolic hormone used to control metabolism and fat homeostasis [1]. Overall, they found significant improvement in myocardial function, FAs oxidation, and electron transport chain activity, and decreased lipid accumulation in these cells, suggesting that Adipsin and Irak2 may be used to treat damaged mitochondrial tissue and alleviate myocardial lipotoxicity in patients with DCM. This research illuminates a novel mechanism of Adipsin alteration in DCM, revealing a potential method to reduce mitochondrial dysfunction.

First, Jiang et al. [2] established how Adipsin induces myocardial protection through interactions with the Irak2 protein in cardiomyocytes. In the mouse model, they found that Adipsin overexpression inhibited Irak2 translocation to the mitochondria, which increased prohibitin (Phb) and optic atrophy protein 1 (Opa1) levels, improving mitochondrial structure and mitochondrial electron transport chain activity. Jiang et al. [2] also induced DCM through a high-fat diet, which mimics only the early stages of diabetes, as the animals do not develop β-cell failure [6]. The combination of streptozotocin and a high-fat diet induces the onset and development into later stages of diabetes, including the organ damage observed in DCM [7]. To account for the pathology of DCM more thoroughly, we suggest that further work confirm the role of Adipsin through this method of inducing diabetes. If Adipsin proves to have a weak impact on myocardial cells in more accurate DCM models, it would not be an effective target for treating cardiac dysfunction in human DCM.

Second, Jiang et al. [2] characterized the role of Irak2 in the Adipsin pathway as a downstream modulator. In myocardial tissue, they found that the *Irak2* knockdown model had significantly improved cardiac function, increased oxidative phosphorylation and ATP production. The subsequent overexpression of Adipsin did not further improve these factors. We believe that it would have been pertinent if the study had investigated the improvements to cardiac function under varying levels of *Irak2* knockdown and with *Adipsin* knockout, to determine if the Adipsin/Irak2 interaction is necessary in this model to produce cardioprotective effects. Additionally, interleukin-1, an inflammatory cytokine, directly regulates Irak2 activity in adipocytes [8]. This pathway may provide an earlier and more efficient target for regulating metabolic activity before ectopic lipid accumulation occurs, because Adipsin did not induce further metabolic improvements following *Irak2* knockdown [2], suggesting that Adipsin’s effects may be limited to specific conditions.

Finally, Jiang et al. [2] examined the primary metabolic pathway affected by diabetes: FAs oxidation. In alignment with previous results, they found that Adipsin overexpression reduced the accumulation of myocardial lipids, triglycerides, and malondialdehydes, and restored FAs oxidation in cardiomyocytes. These results also hint at the possibility of targeting FAs oxidation through Irak2 to restore mitochondrial activity. However, previous studies have shown that targeting upstream of cardiac FAs oxidation to induce metabolic adaptation is also effective in treating the cardiac pathology of DCM [4, 9]. For example, AlTamimi et al. [4] found that the Fisetin compound preserved cardiac function by increasing cardiac glucose metabolism and suppressing protein kinase R (which reduced cardiac inflammation and apoptosis). These studies identified mechanisms upstream of FAs oxidation that prevent ectopic lipid accumulation before it occurs, so the impacts of these treatments vs. Adipsin must be experimentally compared to ensure a robust treatment for DCM.

With few prevention and treatment options, accompanied by very few early symptoms for diagnosis, DCM has become a public health issue that urges researchers to understand its mechanism. Because diabetes is a complex disease with several underlying metabolic processes, the role of Adipsin must be validated throughout various stages of diabetes, and with different underlying etiologies. Research investigating the potential of Irak2 in ameliorating diabetes-deteriorated cardiac function is sparse; we suggest this protein and its upstream effectors be investigated as an additional and possibly more thorough way to treat DCM. These avenues for research, combined with promising findings of Jiang et al. [2], have potential therapeutic applications for targeting lipid accumulation to alleviate myocardial dysfunction and restore mitochondrial structure. These insights may also inform treatments for ectopic lipid accumulation in other organs. Additional research is needed to elucidate the long-term impacts of Adipsin and Irak2 manipulation in humans with DCM, including those serving in the military.

## Data Availability

Data sharing is not applicable to this article as no datasets were generated or analyzed during the current study.

## Abbreviations

DCM: Diabetic cardiomyopathy
FAs: Fatty acids
Irak2: Interleukin-1 receptor-associated kinase-like 2
Phb: Prohibitin
Opa1: Optic atrophy protein 1
